# Effects of Prolonged Dietary Curcumin Exposure on Skeletal Muscle Biochemical and Functional Responses of Aged Male Rats

**DOI:** 10.3390/ijms20051178

**Published:** 2019-03-07

**Authors:** Candace N. Receno, Chen Liang, Donna L. Korol, Mustafa Atalay, Kevin S. Heffernan, Tom D. Brutsaert, Keith C. DeRuisseau

**Affiliations:** 1 201 Women’s Building, Department of Exercise Science, Syracuse University, Syracuse, NY 13244, USA; creceno@syr.edu (C.N.R.); cliang06@syr.edu (C.L.); ksheffer@syr.edu (K.S.H.); tdbrutsa@syr.edu (T.D.B.); 2 107 College Place, Department of Biology, Syracuse University, Syracuse, NY 13244, USA; dlkorol@syr.edu; 3 Yliopistonranta 1 E, Institute of Biomedicine, Physiology, University of Eastern Finland, P.O. Box 1627, 70211 Kuopio, Finland; mustafa.atalay@uef.fi

**Keywords:** sarcopenia, Nrf2, antioxidants, oxidative damage, redox status

## Abstract

Oxidative stress resulting from decreased antioxidant protection and increased reactive oxygen and nitrogen species (RONS) production may contribute to muscle mass loss and dysfunction during aging. Curcumin is a phenolic compound shown to upregulate antioxidant defenses and directly quench RONS in vivo. This study determined the impact of prolonged dietary curcumin exposure on muscle mass and function of aged rats. Thirty-two-month-old male F344xBN rats were provided a diet with or without 0.2% curcumin for 4 months. The groups included: *ad libitum* control (CON; *n* = 18); 0.2% curcumin (CUR; *n* = 18); and pair-fed (PAIR; *n* = 18) rats. CUR rats showed lower food intake compared to CON, making PAIR a suitable comparison group. CUR rats displayed larger plantaris mass and force production (vs. PAIR). Nuclear fraction levels of nuclear factor erythroid-2 related-factor-2 were greater, and oxidative macromolecule damage was lower in CUR (vs. PAIR). There were no significant differences in measures of antioxidant status between any of the groups. No difference in any measure was observed between CUR and CON rats. Thus, consumption of curcumin coupled with reduced food intake imparted beneficial effects on aged skeletal muscle. The benefit of curcumin on aging skeletal muscle should be explored further.

## 1. Introduction

Sarcopenia, which is the decline in muscle mass and functional quality with advancing age, afflicts an average of 5%–13% of those aged 60–70 years and 11%–50% of those older than 80 years of age [1]. The ability to execute basic personal care tasks becomes difficult and there is a greater risk of falls and injury among individuals suffering from sarcopenia [2]. These challenges result from a loss of muscular strength, especially in the lower body [3]. Reports document declines in knee extensor torque and power ranging from 20%–40% in individuals aged 60–86 years when compared to younger counterparts [4,5]. Muscle mass has also been shown to decrease in the aging population, with total appendicular muscle mass losses of ~10%–15% reported for men and women [6]. 

The etiology of sarcopenia is complex, as the aging process is characterized by increased levels of skeletal muscle oxidative stress that can disrupt redox regulation of cellular functions, alter transcription factor activity, and damage proteins, lipids and DNA [7]. The age-related shift in oxidative load can result from increased oxidation, decreased antioxidant response, or a combination of these conditions [7]. For example, an increased production of reactive oxygen and nitrogen species (RONS) in aging skeletal muscle was accompanied by a decline in antioxidant status in human vastus lateralis, suggesting a compromised functional response [8]. RONS formation beyond levels that can be quenched by antioxidants may damage cellular components, compromise cell integrity and function, and disrupt redox regulation of cellular function. Although several key antioxidants in aged muscle display an upregulation compared to levels in younger muscle [9,10,11], the antioxidant response to increases in RONS appears to be compromised [12,13]. 

Nuclear factor erythroid-2 related-factor-2 (Nrf2) is a transcription factor that serves as the “master transcriptional regulator” of antioxidant defenses [14]. During instances of increased reactive oxygen species generation, Nrf2 binds to the antioxidant response element (ARE) and upregulates antioxidant enzyme gene expression [15,16]. Several studies connected aging-associated Nrf2 regulation and antioxidant response impairment with increases in oxidative stress and muscle degeneration [17,18,19]; suggesting that Nrf2 activation is critical for normal functioning of aged skeletal muscle. Increasing the activation of Nrf2 could be a strategy to enhance the antioxidant status of aged skeletal muscle and limit the extent of oxidative stress.

Curcumin, an ingredient in the spice turmeric, has garnered attention due to its ability to alleviate disease pathologies through its anti-inflammatory, anti-carcinogenic and antioxidant properties [20]. The ability of curcumin to alter the cellular redox status could be particularly beneficial to aging muscle. Namely, curcumin can directly quench free radicals [21] and promote the nuclear translocation and activation of Nrf2 through dissociation of Nrf2 from kelch-like ECH-associated protein 1 (Keap1) [22,23]. Studies have documented curcumin’s ability to upregulate Nrf2 in skeletal muscle [24], as well as its overall efficacy in conditions of skeletal muscle wasting, such as sepsis and inflammation [25,26,27]. However, there is limited knowledge of the effectiveness of curcumin on sarcopenic muscle. A recent study reported that healthy individuals over the age of 65 years who supplemented with Meriva^®^, a commercially available form of curcumin, for three months showed greater physical performance compared to baseline measurements [28]. While the specific mechanisms by which curcumin mediates positive effects on sarcopenic muscle are lacking, previous work suggests that curcumin supplementation may be beneficial.

The primary purpose of this study was to determine whether aged F344xBN rats exposed to prolonged dietary curcumin would exhibit greater muscle mass and function compared to controls. Nuclear Nrf2 levels and markers of antioxidant status, in addition to indicators of oxidative stress were examined to identify potential mechanisms that mediate skeletal muscle functional adaptations following prolonged dietary curcumin supplementation in aged rats. Additionally, a small complimentary study was conducted to determine effects of prolonged curcumin exposure, delivered by osmotic pumps, on skeletal muscle mass and function. 

## 2. Results

### 2.1. Food Intake, Body Mass and Muscle Mass

**Figure 1:**
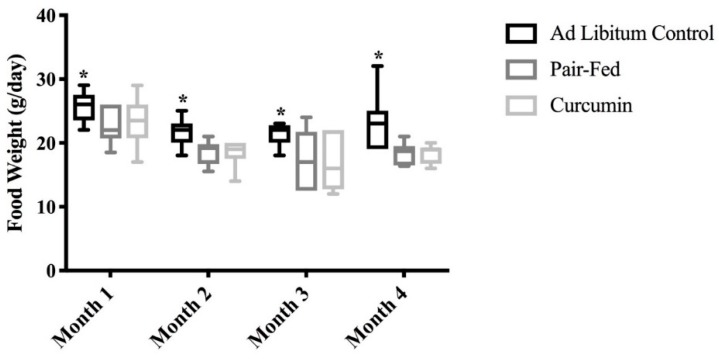
Food weight among the groups (CON, PAIR, & CUR). CON showed greater food intake over the duration of the experiment compared to CUR and PAIR (* *p* < 0.05); Analyses included 1-way ANOVA between feeding groups, box and whisker plots depict 95% confidence interval (CI).

Food intake among CUR and PAIR rats was lower than that of CON rats during each month of the 4-month supplementation period (*p* < 0.05, Figure 1). Body mass values were similar among groups (*p* = 0.991, Table 1). Plantaris muscle mass of PAIR rats was significantly lower than that of the CON and CUR rats (*p* = 0.021 and *p* = 0.011 respectively, Table 1).

### 2.2. Muscle Contractile Function

**Figure 2:**
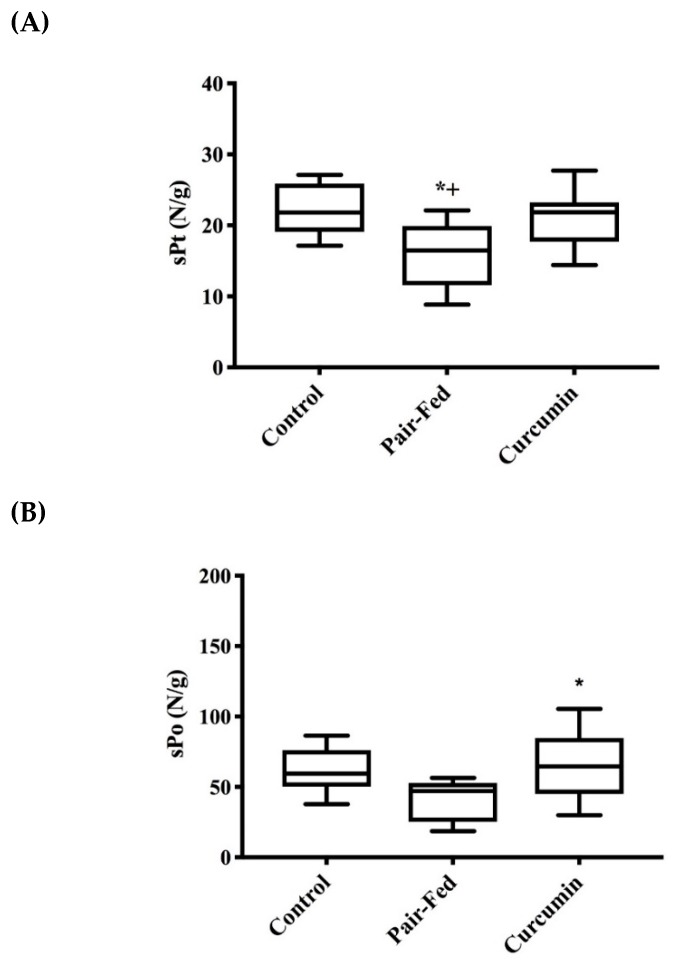
Muscle twitch and tetanic tension at 36 months of age: (**A**) PAIR showed lower peak twitch tension compared to CON (* *p* = 0.013) and CUR (+ *p* = 0.026) (*n* = 8/group); and (**B**) PAIR displayed lower force compared to CUR (* *p* = 0.040, *n* = 8/group). Analyses included 1-way ANOVA, box and whisker plots depict 95% confidence interval (CI). sPt = specific twitch tension, sPo = specific peak tetanic tension.

Plantaris peak twitch tension of PAIR rats was lower when compared to CON and CUR rats (*p* = 0.013 and *p* = 0.026 respectively), but no difference was observed between CUR and CON rats (*p* = 0.817, Figure 2A). Peak tetanic tension was also lower among PAIR rats compared to CUR rats (*p* = 0.040, Figure 2B).

### 2.3. Skeletal Muscle Nuclear Nrf2 Expression & Antioxidant Measures

**Figure 3:**
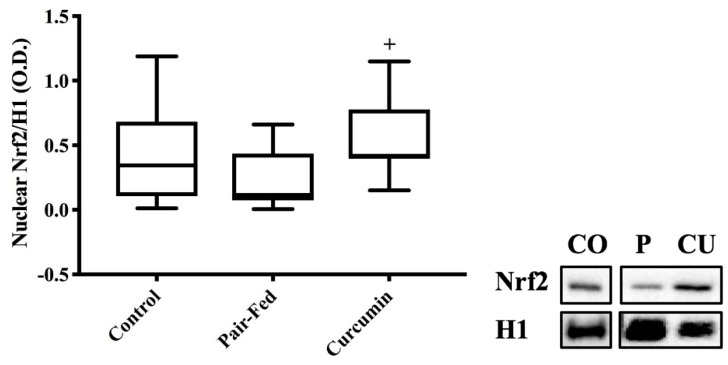
Nuclear factor erythroid-2 related-factor-2 (Nrf2) protein expression at 36 months of age. Expression was greater in CUR compared to PAIR (+ *p* = 0.008, *n* = 9/group). Representative images for Nrf2 and Histone-1 (H1) are shown; CO = CON, P = PAIR, CU = CUR. Bands are separated in instances when samples were not adjacent on same membrane. Analyses included 1-way ANOVA, box and whisker plots depict 95% confidence interval (CI).

The CUR rats showed greater nuclear Nrf2 levels compared to PAIR rats (*p* = 0.008, Figure 3). There were no differences in any of the plantaris antioxidant measures, including catalase expression/activity, manganese superoxide dismutase (MnSOD) expression/activity, heme-oxygenase-1 (HO-1) expression, thioredoxin/thioredoxin-interacting-protein (TRX/TxNIP) ratio or total antioxidant capacity between groups (*p* > 0.05, Table 2). 

### 2.4. Skeletal Muscle Oxidative Stress 

**Figure 4:**
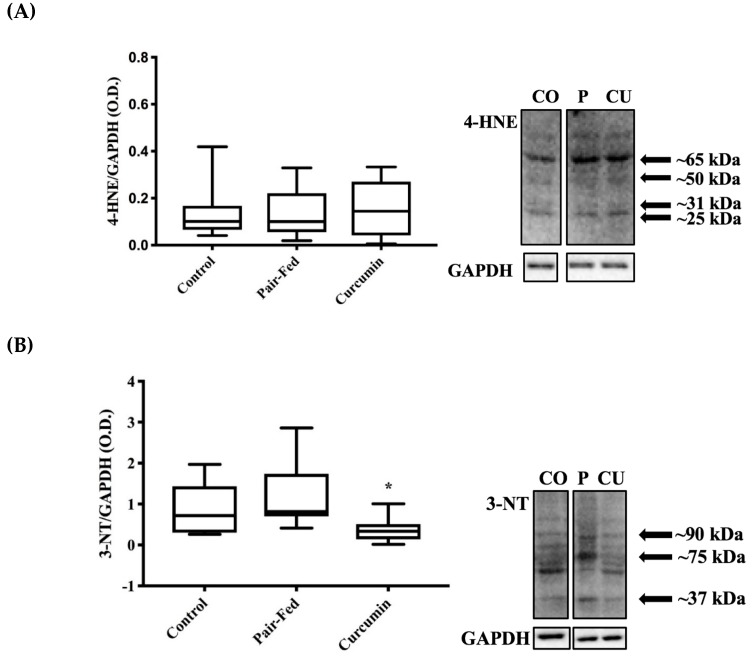

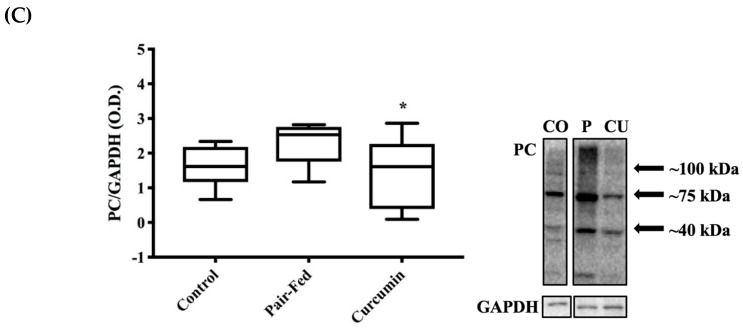
Oxidative damage markers at 36 months of age (CON, PAIR, & CUR): (**A**) 4-hydroxynonenal (4-HNE) adducts, no significant differences were observed (*p* = 0.935, *n* = 9/group); (**B**) CUR showed lower 3-nitrotyrosine (3-NT) compared to PAIR (* *p* = 0.035, *n* = 9/group); (**C**) CUR displayed lower levels of protein carbonyls (PC) compared to PAIR (* *p* = 0.042, *n* = 9/group); Representative images for 4-HNE adducts, 3-NT, and PC are shown; CO = CON, P = PAIR, CU = CUR. Bands are separated in instances when samples were not adjacent on same membrane. Arrows indicate prominent bands that were used for evaluation. Analyses included 1-way ANOVA, box and whisker plots depict 95% confidence interval (CI).

The level of skeletal muscle 4-hydroxynonenal (4-HNE) adducts was similar among the animal groups (*p* = 0.935, Figure 4A). However, 3-nitrotyrosine (3-NT) and protein carbonyls (PC) levels, which are indicative of oxidative damage and modifications to proteins, were significantly lower among CUR compared to PAIR (*p* = 0.035 and *p* = 0.042 respectively, Figure 4B,C). 

### 2.5. Curcumin Administration via Osmotic Pumps: Complementary Study

No significant difference in body mass or food consumption was observed between control and curcumin groups (*n* = 3/group) (*p* > 0.05). Notably, rats provided with curcumin showed greater plantaris mass (0.28 (0.02) vs. 0.22 (0.01) g, curcumin vs. control; *p* = 0.012). There was an apparent greater specific peak twitch (20.5 (8.9) vs. 16.1 (11.5) N/g, curcumin vs. control) and specific tetanic (65.2 (27.4) vs. 43.6 (22.8) N/g curcumin vs. control) tension response of the plantaris from curcumin supplemented animals. Differences in force production measures, however, did not reach statistical significance.

## 3. Discussion

The primary aim of this study was to document the effects of prolonged dietary curcumin exposure on aged skeletal muscle using an animal model that reflects the time course of human sarcopenia. Rats displayed notable differences between the curcumin and pair-fed groups that included larger muscle mass and greater force production. Additionally, greater nuclear levels of Nrf2 and lower oxidative protein damage were observed in curcumin-fed animals. However, no difference in any measure was observed between CUR and CON rats, suggesting that curcumin did not impart beneficial effects compared to *ad libitum* feeding with a normal diet. A discussion of these findings is presented in the following sections.

### 3.1. Rats Consuming Curcumin Showed Reduced Food Intake 

We made use of a 0.2% curcumin (by food weight) intervention, which is similar to previous rat studies investigating efficacy of curcumin on a variety of tissues, including liver, adipose, and skeletal muscle [29,30,31,32,33]. This supplementation amount resulted in an average daily curcumin intake of ~77 mg, similar to what was reported by another study examining curcumin effects on liver and adipose tissue [33]. While curcumin’s bitter taste has been acknowledged in the literature [34,35], a lower food intake among curcumin-fed rats has not been reported previously. However, since previous dietary curcumin supplementation studies involved younger animals [30,36] the current data are the first to show reduced food intake in aged rats. The lower food intake among CUR rats is suggestive that aged rats may be more sensitive to the bitterness of curcumin. Thus, the difference in food intake between the CUR and CON groups underscores the importance of including a pair-fed group in this study. As a result, we considered the suitable comparison group for the CUR rats to be the PAIR animals, which were matched for food intake throughout the duration of the 4-month supplementation period. The average daily food intake amounts and body weights for all animals were within ranges reported previously for male F344xBN rats [37]. 

### 3.2. Exposure to Dietary Curcumin Altered Muscle Mass, but not Body Mass 

Prolonged curcumin exposure did not alter the body mass of rats, which is a finding in agreement with previous studies employing a rat model of curcumin supplementation ranging in time from 2 to 16 weeks [30,38,39]. However, dietary curcumin exposure positively impacted muscle mass of CUR rats compared to pair-fed controls. The greater skeletal muscle mass of CUR rats is consistent with previous curcumin supplementation studies, in which curcumin administration spared muscle mass across different models of muscle wasting through both an attenuation of oxidative stress and inflammation [25,40]. Notably, pair feeding of aged rats resulted in decreased food intake as well as lower muscle mass compared to the normal *ad libitum* fed group. While caloric restriction is generally thought to elicit beneficial adaptations when nutrition is balanced [41], metabolic processes such as protein turnover are compromised with a general decrease in food intake [42]. Thus, the lower muscle mass of the aged PAIR animals is consistent with previous reports involving food restriction, in which lean body mass is negatively affected [43]. Interestingly, studies suggest decreased food intake among rats is associated with increased activity levels; experimentally imposed food restriction has led to augmented wheel running [44]. However, these studies utilized a more pronounced food restriction that mirrors anorexia nervosa, whereby rats were only provided 15 g of food daily [45]. As previously noted, food consumption of all groups was within normal ranges previously reported for F344xBN rats at a similar age [37] and the reduction in food intake did not reach levels utilized in food restriction studies. However, it is apparent that the difference in food intake was large enough to impact muscle mass and function. Overall, dietary curcumin exposure appeared to maintain muscle mass when food intake was restricted, but did not impart beneficial effects on muscle mass and function compared to rats that were allowed *ad libitum* access to food. Whether curcumin administration under *ad libitum* conditions can augment muscle mass remains to be determined and is a topic addressed by our complimentary study (discussed below). 

### 3.3. Curcumin Exposure Effects on Nuclear Nrf2 Protein Expression and Antioxidant Responses 

Previous reports suggest that Nrf2 activation is impaired in aged skeletal muscle that is associated with increased oxidative stress and compromised antioxidant capacity [46,47]. Thus, upregulation of Nrf2 is a potential means by which curcumin may exert beneficial effects on aged muscle. The presence of greater Nrf2 nuclear levels in CUR muscle suggests that curcumin supplementation augmented Nrf2 translocation in response to pair feeding. This finding is of utmost importance, as Nrf2 modulates the expression of numerous genes including those related to antioxidant expression, protein stability, and inflammation [48]. A study by He et al. (2012) reported greater muscular Nrf2 activation after 15 days of curcumin supplementation in a mouse model of high fat diet induced insulin resistance compared to controls [49]. Previous studies implementing shorter supplementation periods also reported Nrf2 upregulation in the brain and liver of rodents [50,51]. Hence, our findings add to the body of literature showing the ability of the Nrf2 pathway in skeletal muscle to respond positively to curcumin supplementation.

Although we hypothesized a greater nuclear Nrf2 level in response to CUR would result in an increased antioxidant response, no changes to any of the antioxidants studied were detected. These data suggest that even with curcumin supplementation mediating Nrf2 translocation, a dysfunction in the ability of antioxidants to respond in aged muscle remained. Previous studies that reported curcumin-induced alterations in antioxidant expression used supplementation durations on the order of hours to days [52,53,54], suggesting that exposure duration may be an important consideration. In this regard, it is possible that the 4-month period was too long to capture short-term changes in antioxidants. When provided for 2–4 days, curcumin increased HO-1 expression in rats with liver injuries [52]. Similarly, human studies examining antioxidant expression after damage-inducing exercise reported total antioxidant capacity and catalase activity were only increased up to several hours post-supplementation, while other antioxidants were unchanged [53,54]. 

The idea of a blunted antioxidant response may explain the findings of the current study, as greater nuclear Nrf2 levels in aged CUR did not occur in conjunction with differences in antioxidant levels. CUR rats may have experienced an antioxidant blunting effect possibly due to old age, or antioxidant expression changes were short-lived; possibly explaining the lack of observable changes in the antioxidant response noted after 4 months of curcumin exposure. Transient changes in antioxidant defenses were characterized in the human muscle disuse model, where expression of HO-1, glucose-regulated protein-75 (Grp75), and glutathione levels increased at varying time points during bed rest; prior to the development of oxidative stress [55]. Alternatively, since Nrf2 is a transcription factor that can mediate various responses [14], it is possible that other pathways were altered by CUR exposure. 

Nrf2 activation may decrease the skeletal muscle inflammatory response through suppression of nuclear factor-kappa B (NF-κB), a pro-inflammatory transcription factor [56]. Interestingly, markers related to NF-κB activation were shown to be upregulated in aged skeletal muscle [57,58], but the mechanisms underlying NF-κB’s action in sarcopenia are poorly understood. While studies report inhibitory actions of curcumin on NF-κB in other models of skeletal muscle wasting, the mechanistic actions of curcumin in aging muscle may be distinct from those involved in necrosis, disuse and exercise-induced muscle damage [29,40]. For example, while inhibition of NF-κB improved insulin resistance in aged mice, it also resulted in decreased muscle mass and measures of force production [59]. Cachexia also results in NF-κB mediated muscle wasting [60], which may explain why curcumin supplementation improved muscle responses in conjunction with food restriction. However, due to the comprehensive role of NF-κB in skeletal muscle, possible inhibition of NF-κB may also explain why curcumin supplementation did not lead to improved skeletal muscle mass and function above what was observed in control *ad libitum* fed rats. Further evaluation of NF-κB in aging skeletal muscle, in conjunction with curcumin-mediated Nrf2 activation, would be beneficial in solidifying the role of NF-κB in sarcopenia and the potential for curcumin to alter this pathway. 

### 3.4. Curcumin Exposure and Muscle Oxidative Damage 

Muscle functional differences between CUR and PAIR rats mirrored the difference in plantaris muscle mass, even after force production values were normalized. The greater tetanic tension obtained in curcumin-fed aged rats is consistent with a study that investigated the effects of curcumin on hindlimb unloading-induced muscle wasting. In this report, soleus muscle of curcumin supplemented animals displayed increased tetanic tension and larger muscle mass compared to control muscle [61]. The imposed food restriction was associated with greater levels of oxidative damage in PAIR compared to CUR rats. Thus, our findings suggest the greater muscle mass and functional response of CUR rats may be related to lower oxidative stress. The ability of curcumin to counteract oxidative stress in skeletal muscle is well documented [25,49,54,61,62] and the difference in protein oxidative damage may be a potential mechanism by which curcumin intake was associated with greater muscle mass and force production. For example, oxidative stress can cause muscle contractile dysfunction by impairing calcium regulation and calcium channel activity via modification of ryanodine receptors [8,63]. Curcumin-induced increases in Nrf2 activity were previously associated with declines in oxidative stress in skeletal muscle mitochondria, a major source of oxidant production and dysfunction during aging [64]. It is possible that the oxygen radical scavenging ability of curcumin played a direct role in quenching RONS [65,66] that may also explain the lack of changes to antioxidant enzymes and lower level of oxidative damage markers. However, despite the lack of change in the TRX/TXNip ratio, which provides insight into the thiol antioxidant response, assessment of the glutathione system could provide further insight into the potential impact of curcumin on skeletal muscle redox status regulation [67,68]. Collectively, while prolonged exposure to curcumin prevented food restriction effects on aged skeletal muscle, the response of CUR rats was not different from values of rats with *ad libitum* food intake. 

### 3.5. Curcumin Administration to Aged Rats via Osmotic Pump Results in Greater Skeletal Muscle Mass

The palatability of curcumin likely resulted in the food intake difference between the CON and CUR groups. Thus, the benefits of curcumin in this study were observed under the condition of food restriction. While these results are relevant to a specific subset of the human population that experiences food restriction, the efficacy of curcumin supplementation under *ad libitum* food intake conditions was still undetermined. To address this point, we collected data from a small cohort of rats that were provided curcumin via a subcutaneous osmotic pump, a method previously used for administration of curcumin and for delivery of compounds to aged rats [69,70,71,72]. Results of the study revealed that 4-months of subcutaneous curcumin administration resulted in a significantly greater plantaris mass (*n* = 3/group). There was also an approximate 25% greater twitch and ~30% greater tetanic tension, but these variables did not reach statistical significance. Importantly, these curcumin-induced responses were observed under the same dietary conditions, suggesting that curcumin may impart benefits beyond what is observed with normal feeding. Future studies may consider the use of osmotic pumps to avoid curcumin feeding issues that may arise when working with aged rats.

### 3.6. Limitations

While this study was successful in determining the effects of dietary curcumin exposure on skeletal muscle, we acknowledge some limitations within our experimental design. Since the goal of this study was to document effects of long-term exposure to dietary curcumin, measures of interest were obtained following 4 months of supplementation. The data do not depict changes (particularly in antioxidant response) that may be transient throughout this period. Moreover, numerous reports suggest that curcumin may work through various pathways. For example, NF-κB signaling may have been altered by curcumin but was not assessed in this study. Additional mechanisms (e.g., apoptosis, protein and insulin signaling) are also relevant to aging muscle but were outside the scope of this study. The presence of curcumin at the tissue or systemic level was not assessed, which is a study limitation that could have contributed to variability observed in our measures. The specific dosage of curcumin used in this study was based on previous studies that showed responses in rat skeletal muscle and other tissues [29,30,31,32,73].

This study employed dietary curcumin supplementation to male rodents beginning at 32 months of age, a time at which muscle mass has already begun to decline. Our study highlights the role of curcumin as an intervention for sarcopenia. We focused on males because of their larger decrement in muscle mass and function at the defined age range of this study [74]. Thus, our findings uncover an ability of curcumin to act within a population greatly susceptible to muscle atrophy and dysfunction. However, understanding the role of curcumin in modulating the muscle redox balance of the sarcopenic female population should also be documented to understand the efficacy of curcumin for the sarcopenic population as a whole. 

## 4. Materials and Methods

### 4.1. Ethical Approval

Use of animals was in accordance to the policies described in the Guide for the Care and Use of Laboratory Animals [75]. All procedures were submitted to and approved by the Syracuse University Institutional Animal Care and Use Committee (Project identification code: 15-007; Date of approval: 22 September 2015).

### 4.2. Animals

Thirty-two-month-old male F344xBN rats were obtained from the National Institutes of Health, National Institute on Aging, Aged Rodent Colony. Upon arrival to the Syracuse University animal care facility the rats were singly housed in Optirat cages (Animal Care Systems, Centennial, CO, USA) under standard laboratory environmental conditions (12:12 light/dark cycle, 40% to 60% humidity and 20–25 °C) and provided access to food and water *ad libitum*. 

### 4.3. Experimental Design 

Three groups of F344xBN male rats (thirty-two-month-old, *n* = 18/feeding group) were used in these experiments. Rats were randomly subdivided into the following three groups: *Ad libitum* normal diet (CON); *ad libitum* curcumin supplemented diet (CUR); and pair-fed normal diet (PAIR). The CUR group received a similar purified diet to that of the PAIR and CON (AIN93M; Teklad Diets, Madison, WI, USA) groups but was supplemented with 0.2% curcumin (by food weight), as Turmeric Type 97 (kindly provided by Kalsec Inc., Kalamazoo, MI, USA). Rats were on respective diets for a period of 4 months and food consumption was assessed daily. Each PAIR rat was randomly matched with a CUR rat. The weekly food intake of CUR rats was provided to the corresponding PAIR rats one week later. Thus, the PAIR rats were on a feeding schedule that was one week behind the CUR rats. To ensure similar food intake, PAIR rats were fed on a daily basis using the average amount of food per day of the matched CUR rats. PAIR rats were included since a reduced food intake was anticipated among CUR rats, due to the bitter taste of curcumin. After the 4-month supplementation period the rats were anesthetized and assessed for in situ muscle contractile function followed by tissue collection. Rats were humanely euthanized following the experiments by an intracardiac injection of Fatal Plus (50 mg/kg; Vortech Pharmaceuticals Ltd., Dearborn, MI, USA). A gross necroscopy was conducted to document the presence of tumors, lesions, or other signs of illness. 

### 4.4. Plantaris Muscle Contractility

On the day of contractile recordings, rats were weighed and anesthetized with an i.p. injection of Fatal Plus (80–120 mg/kg), which was administered as needed throughout the remainder of the muscle function experiment. Anesthetic depth was monitored throughout the recording session using both corneal reflex and toe-pinch response. Once in a surgical plane of anesthesia, rats were placed on a heating blanket attached to a rectal thermistor to maintain body temperature at approximately 37 °C. A surgical procedure was performed to expose the femur and isolate and incise the sciatic nerve near the lateral, distal portion of the right leg. The right plantaris muscle was surgically isolated and 2–0 silk suture was threaded through the distal tendon of the muscle. Extreme care was taken to ensure that the blood supply to the muscle remained intact. The rat was then transferred to a heated platform (806D in situ rat apparatus, Aurora Scientific Inc., Aurora, ON, Canada) in the prone position and the right leg was secured using conical screws placed at the distal femur and tibia. The suture was fastened to the arm of the force transducer (305C-LR, Aurora Scientific Inc., Aurora, ON, Canada). The plantaris muscle surface temperature was maintained at approximately 35 °C by shining a lamp on the muscle and routinely applying a warmed mineral oil/Vaseline mixture.

Force output was recorded via a computerized data-acquisition system (DMA v4.1.6; Aurora Scientific Inc., Aurora, ON, Canada). The muscle was stimulated with platinum wire electrodes placed at the sciatic nerve using supramaximal monophasic pulses of 0.5 ms. The optimal contractile length was determined by systematically lengthening the muscle and evoking single twitches. After the muscle was stimulated to contract, the observed force output was recorded. The muscle length was increased to a degree that elicited a 0.2 g increase in resting tension. This process was repeated until a plateau in muscle force was observed. Thereafter, all contractile properties were measured isometrically at optimum contractile length. Maximal twitch and tetanic tension (120 Hz, 300 ms trains) were recorded, with contractions separated by a 2-min recovery period. Raw contractile force measurements (N) are expressed per gram of plantaris muscle mass.

### 4.5. Tissue Collection

At the end of the muscle function recording session the right and left plantaris muscles were carefully removed, rinsed in 0.9% NaCl and gently wiped with a cotton swab to remove blood and the mineral oil/Vaseline mixture. The right plantaris was trimmed of excess connective tissue, weighed, and cut at the mid-belly; one-half of the muscle was embedded in OCT and frozen in liquid nitrogen. The remaining tissue, and left muscle, was frozen in liquid nitrogen for biochemical analyses. 

### 4.6. Western Blot 

Plantaris muscle was homogenized (1:10, *w*:*v*) in RIPA lysis buffer (Santa Cruz Biotechnology, Dallas, TX, USA) and centrifuged at 10,000× *g* for 10 min at 4 °C. The protein content of the soluble fraction was assessed using the RC DC Protein Assay (Bio-Rad, Hercules, CA, USA). Proteins (~50 μg) were individually separated by SDS-PAGE and transferred to nitrocellulose membranes via iBlot Transfer System (Thermo Fisher Scientific, Waltham, MA, USA). Membranes were subsequently blocked (1%–5% using skim milk protein in PBS containing 0.05% Tween-20) followed by incubation with a primary antibody directed against 4-hydroxynonenal adducts (4-HNE) (Alpha Diagnostic International, San Antonio, TX, USA; RRID:AB_2629282) [76], 3-nitrotyrosine (3-NT) (Alpha Diagnostic International; RRID:AB_1620181) [77,78], manganese superoxide dismutase (MnSOD) (Cayman Chemical, Ann Arbor, MI, USA; RRID:AB_1213308) [79,80], catalase (GeneTex, Irvine, CA, USA; RRID:AB_10726597) [81,82], heme-oxygenase-1 (HO-1) (Enzo Life Sciences, Farmingdale, NY, USA; RRID:AB_2039226) [83,84], thioredoxin (TRX) (IMCO, Sweden; RRID:AB_2725744) [85,86] or thioredoxin-interacting-protein (TxNip; MBL International, Woburn, MA, USA; RRID:AB_592934) [87] diluted in blocking buffer. This was followed by incubation with a horseradish peroxidase–antibody conjugate (1:1000-1:5000) directed against the primary antibody for 1hr at RT. The membranes were treated with chemiluminescent reagents (Bio-Rad) and imaged using a ChemiDoc XRS+ System and Image Lab Software (Bio-Rad). Protein is expressed relative to a loading control protein, glyceraldehyde 3-phosphate dehydrogenase (GAPDH) (Cell Signaling Technology Danvers, MA, USA; RRID:AB_561053) [88,89] or β-actin (Santa Cruz Biotechnology; RRID:AB_626632) [90,91].

### 4.7. Protein Carbonyls (PC) 

Protein oxidative damage was assessed by the measurement of PC, as previously described [92]. Derivatization solution consisted of 10× 2,4-dinitrophenylhydrazine dissolved in 100% trifluoroacetic acid (TFA) diluted to a 1× solution within 72 h of use. A neutralization solution was made prior to derivatization that consisted of 2 M Tris/30% glycerol. Protein (20 µg) was derivatized, neutralized, and separated by SDS-PAGE and transferred to nitrocellulose membranes as described in the section above. Membranes were treated with an anti-DNPH primary antibody from a commercial kit (1:1000) (Millipore, Billerica, MA, USA) followed by a horseradish peroxidase-antibody conjugate directed against the primary (anti-rabbit; 1:1000). The membranes were imaged for analysis following application of chemiluminescent reagents as described above.

### 4.8. Nuclear Protein Fractions for Nrf2

Plantaris muscle was homogenized using a glass-on-glass homogenizer in ice-cold cell lysis buffer (10 mM NaCl, 1.5 mM MgCl_2_, 20 mM Hepes, pH 7.4, 20% glycerol, 0.1% Triton X-100, and 1 mM DTT) at 4 °C. Homogenate was centrifuged for 5 min at 3000× *g* at 4°C, and the pellet was resuspended in buffer (20 mM HEPES, pH 7.9, 25% glycerol, 500 mM NaCl, 1.5 mM MgCl_2_, 0.2 mM EDTA) prior to undergoing an additional 5 min centrifugation at 3000× *g*. The supernatant was transferred to a 10K nominal molecular weight limit filter unit (EMD Millipore, Bilerca, MA, USA) with an equal volume of binding buffer (40 mM HEPES, pH 7.9, 10% glycerol, 3 M KCl, 500 mM MgCl_2_, 0.5 mM DTT) and centrifuged at 3000× *g* for 30 min at 4 °C. Binding buffer was added to the filter tube and centrifugation was repeated. The resulting nuclear fraction was measured for protein concentration via the Bradford method and prepared for western blotting as described above using a primary antibody (1:1000) directed against Nrf2 (Santa Cruz Biotechnology; RRID:AB_1125852) [93]. Nuclear Nrf2 expression was normalized to histone-1 protein (H1) as a control (Santa Cruz Biotechnology; RRID:AB_675641) [94,95,96,97,98].

### 4.9. Muscle Antioxidant Capacity

Skeletal muscle antioxidant capacity was assessed using the commercially available Antioxidant Assay Kit (Cayman Chemical). Plantaris muscle was homogenized in buffer (5 mM potassium phosphate, pH 7.4, 0.9% NaCl) and centrifuged at 10,000× *g* at 4 °C for 15 min. Sample (10 µL) was added to 10 µL metmyoglobin and 150 µL of chromogen. Reactions were initiated with 40 µL of hydrogen peroxide working solution. After 5 min of incubation the samples were read at 750 nm using a spectrophotometer (Powerwave HT, BioTek Instruments Inc., Winooski, VT, USA). Values are expressed as mM/mg protein. 

### 4.10. Catalase Activity

Catalase activity was assessed using a modified assay [99]. Plantaris muscle was homogenized in ice-cold 100 mM potassium phosphate buffer (pH 7.4) with 0.05% BSA in dH_2_O. The homogenate was centrifuged at 400× *g* for 10 min at 3 °C. The resulting supernatant was vortexed with ethanol and incubated for 30 min on ice. After incubation, 1% Triton X-100 was added to the homogenate, vortexed, and incubated on ice for 15 min. Homogenate (150 μL) was added to 1050 μL of 10 mM H_2_O_2_ and read by a spectrophotometer at 240 nm for 1 min. Readings were compared to a blank control containing 150 µL homogenate with 1050 µL of 100 mM potassium phosphate buffer. The decomposition of H_2_O_2_ detected as decreases in absorption was used as the measure of catalase activity. The follow equation was used to calculate K/mg protein:

K = (2.3/t) × [log_10_ (A1/A2)] × DF

where t = time in minutes; A1 = initial absorbance reading − blank absorbance reading; A2 = absorbance reading at 1 min − blank absorbance reading at 1 min; DF = dilution factor of assay

### 4.11. Manganese Superoxide Dismutase (SOD) Activity

MnSOD activity was measured using a commercially available assay kit (Sigma-Aldrich, St. Louis, MO, USA). Muscle was homogenized in buffer (50 mM Tris-HCl, pH 7.5, 5 mM EDTA and 1 mM DTT) and centrifuged at 10,000× *g* for 15 min at 4 °C. The resulting supernatant (20 µL) was added to 200 µL of Working Solution and 20 µL of Enzyme Working Solution (Sample) or 200 µL of WST Working Solution and 20 µL of dilution buffer (Blank 2). Super clean H_2_O (20 µL) was added to 200 µL of WST working solution and 20 µL Enzyme Working Solution (Blank 1) or 20 µL dilution buffer (Blank 3). After 20 min incubation at RT, samples were read at 450 nm using a spectrophotometer (Powerwave HT, BioTek Instruments Inc.). MnSOD activity was assessed by pre-incubating the homogenate with potassium cyanide. Activity (units/mg protein) was calculated using the following equation:

% inhibition = {([Ablank 1 − Ablank3] − [Asample – Ablank2])/[Ablank1 − Ablank3]} × 100

where A = absorbance

### 4.12. Complimentary Study: Administration of Curcumin via Subcutaneous Osmotic Pumps

Following the collection of muscle functional and biochemical data, we conducted a small complimentary study to investigate the muscle mass and contractile response to an alternative mode of curcumin administration. Two groups of 32-month-old male F344xBN rats (*n* = 6/group) were implanted with subcutaneous pumps containing either 4 mM curcumin in DMSO or DMSO vehicle in the upper back region for a 4-month period of time. Osmotic pumps (2ML4; Alzet, Minneapolis, MN, USA) were replaced every 28 days. Following the curcumin administration period, muscle mass and peak contractile function of the plantaris were obtained as described above.

### 4.13. Statistical Analysis 

Outcome variables were analyzed using 1-way ANOVA, with significance set *a priori* at *p* < 0.05. Analyzed variables included contractile force, nuclear Nrf2 expression, antioxidant capacity and antioxidant activities, markers of oxidative stress, muscle mass, and whole body mass. Independent samples t-tests were used to identify potential differences in plantaris muscle mass between groups in the complimentary subcutaneous pump study, but the small sample size did not allow for detecting changes in contractile measures. Significant differences were identified using Bonferroni corrections for post hoc analysis. Values were only excluded from statistical analysis if they met the following criteria: data were obtained from a rat that did not survive the entire 4-month supplementation protocol and/or the value was determined to be an extreme outlier, as indicated on SPSS generated boxplots. Normality was determined by Shapiro-Wilkins test, and if it was violated (*p* < 0.05) a Box-Cox transformation was applied to meet assumptions of normality. Each transformation was visually checked to ensure graphical patterns were similar to the non-transformed data. Non-transformed data are shown in the figures and tables. IBM SPSS Statistics 25, MiniTab 18, and Prism 6 (GraphPad Software, La Jolla, CA, USA) were used for statistical analyses. Data are reported as mean (SD).

## 5. Conclusions

The goal of this study was to reveal the impact of prolonged exposure to dietary curcumin on aged skeletal muscle. These findings can help build the foundation for determining the efficacy of long-term curcumin supplementation for the treatment of sarcopenia. The ability of curcumin feeding to result in greater mass and function during a food-restricted state shows its potential relevance to particular conditions, such as those leading to cachexia. Moreover, our preliminary findings utilizing subcutaneous pumps further illustrate the potential for curcumin to benefit aging skeletal muscle. Future work should expand upon these findings and continue to determine the efficacy of curcumin on muscle mass and function by employing different populations and modes of administration. 

## Figures and Tables

**Table 1:** Body Mass and Plantaris Muscle Mass.

	Body Mass (g)	Plantaris Muscle Mass (g)
CON	506.4 (51.1)	0.285 (0.056)
PAIR	503.7 (73.5)	0.198 (0.046) *
CUR	504.1 (95.6)	0.326 (0.060)

Body and plantaris muscle mass at 36 months of age (CON, PAIR, & CUR). * Significantly different from CON (*p* = 0.021) and CUR (*p* = 0.011). No differences between groups for body mass (*p* = 0.991). Values are mean (SD).

**Table 2:** Antioxidant status.

	CON	PAIR	CUR	*p*-Value
**Protein Expression**				
Catalase	1.08 (0.83)	0.83 (0.53)	1.03 (0.35)	0.44
MnSOD	2.13 (1.21)	1.96 (0.93)	2.64 (1.52)	0.23
HO-1	2.38 (1.84)	3.42 (1.33)	3.28 (1.42)	0.17
TRX/TxNip	0.52 (0.48)	0.67 (0.60)	1.11 (0.84)	0.34
**Activity**				
Catalase	0.16 (0.07)	0.18 (0.10)	0.18 (0.07)	0.79
MnSOD	3.96 (1.34)	3.12 (1.65)	4.56 (1.57)	0.40
TAC	0.21 (0.10)	0.23 (0.10)	0.22 (0.07)	0.88

Plantaris antioxidant status at 36 months of age (CON, PAIR, & CUR). Catalase, manganese superoxide dismutase (MnSOD), heme-oxygenase-1 (HO-1), and thioredoxin/thioredoxin-interacting-protein (TRX/TxNip) ratio protein expression (O.D); catalase (K/mg protein) and MnSOD (units/mg protein) activity; and total antioxidant capacity (TAC; mM/mg protein). Values are mean (SD).
